# Prevalence and risk factors for viral exposure in rural dogs around protected areas of the Atlantic forest

**DOI:** 10.1186/s12917-016-0646-3

**Published:** 2016-01-28

**Authors:** Nelson Henrique de Almeida Curi, Rodrigo Lima Massara, Ana Maria de Oliveira Paschoal, Amanda Soriano-Araújo, Zélia Inês Portela Lobato, Guilherme Ramos Demétrio, Adriano Garcia Chiarello, Marcelo Passamani

**Affiliations:** Postgraduate Programme in Applied Ecology, Department of Biology, Federal University of Lavras, Lavras, Brazil; Postgraduate Programme in Ecology, Conservation and Management of Wildlife, Department of Biology, Institute of Biological Sciences, Federal University of Minas Gerais, Belo Horizonte, Brazil; Laboratory of Viral Diseases, Department of Preventive Veterinary Medicine, School of Veterinary Medicine, Federal University of Minas Gerais, Belo Horizonte, Brazil; Department of Biology, University of São Paulo, Ribeirão Preto, Brazil

**Keywords:** Atlantic Forest, *Canis familiaris*, Epidemiology, Virus exposure, Risk factor, Human/wildlife interface

## Abstract

**Background:**

Despite the crucial role of domestic dogs as reservoirs for zoonosis and some of the most threatening diseases for wild carnivores such as distemper and parvovirosis, little is known about the epidemiological features and the risk factors involved in pathogen exposure of dogs that live in human/wildlife interfaces and actually contacts wildlife. Through a cross-sectional serological approach and questionnaire survey, we assessed the prevalence along with individual and environment-associated risk factors for four important viral diseases of rural dogs living in households around six Atlantic Forest fragments in southeast Brazil.

**Results:**

Widespread exposure to canine parvovirus (97 %), canine distemper virus (15 %) and canine adenovirus (27 %) was detected, but none for canine coronavirus. Dogs from small private reserves were more exposed to parvovirus and canine distemper virus than those from larger state parks. Exposure was associated with dog sex and age, lack of health care and the number of people in the households. Remarkably, factors linked to free-ranging behaviour of dogs were associated with the exposure for all pathogens detected.

**Conclusions:**

According to identified associations, reducing viral pathogen exposure in dogs will require inhibiting dog’s movements and access to nearby forests and villages and improving veterinary assistance. Promoting dog vaccination and population control through sterilization around protected areas is also necessary. The study provides support for preventive management actions aimed to protect the health of rural dogs, and consequently of Atlantic Forest’s wild carnivores.

## Background

The domestic dog (*Canis familiaris*) is beyond question man’s closest animal species, and consequently the most abundant and widespread carnivore mammal in the world. As such, it represents a conservation problem [1]. Notwithstanding the direct negative impacts on wildlife such as predation, competition and harassment [2], dogs are also the most important reservoirs of diseases that affect wild carnivore species [3]. The furthermost important diseases transmitted from dogs to wild carnivores (rabies, distemper and parvovirosis) are named as “The Big Three” due to the strongly negative impact over the latter populations [3].

Infectious disease-driven mortality is one of the major causes of population decline and extinction of wild mammal carnivores worldwide [1, 3]. It can act in conjunction with other endangerment factors, particularly when populations are small or declining due to habitat loss and fragmentation, or when spill-over occur from sympatric man-subsidized dog populations [3]. As examples, African lions (*Panthera leo*) in the Serengeti ecosystem were continuously threatened by epidemics of canine distemper acquired from dogs and other wild species [4], Ethiopian wolves (*Canis simensis*) were heavily affected by dog-transmitted rabies and distemper [5], and grey wolf (*Canis lupus*) populations were impacted by long-term pup mortality due to parvovirus infection [6] in North America. Although evidence on the role of domestic dogs as reservoirs of disease for wild carnivores is becoming a global pattern with other examples coming from Europe [7], Africa [8], South America [9], and Asia [10], few studies have uncovered canine disease-associated risk factors (e.g.) [9, 11], especially at wildlife/human/domestic animal interfaces, which are predicted hotspots for interspecies pathogen transmission leading to disease induced wildlife mortality and disease emergence [12]. Assessing risk factors for diseases in dog populations would, therefore, shed light on transmission and persistence patterns, being of great value for the directing of disease prevention or control efforts for domestic animals, wildlife and humans [13].

In South America, similarly to what occur in other parts of the world, recent case reports and studies revealed that dogs are sources of dangerous infectious agents such as distemper virus to wild carnivores [9, 14, 15], and that several wild carnivore populations have already been exposed to canine pathogens such as parvovirus, distemper virus, adenovirus, and *Toxoplasma gondii*, among others (e.g.) [16–19]. Fortunately, some studies were also concerned with the detection and estimation of pathogen prevalence in sympatric domestic dog populations in a conservation context (i.e. those living around protected areas) [18–23]. Dogs interact with wildlife, and the strength of such interactions is mediated by the role of the dog in the household and the level of care and nutrition given by their owners [24]. However, determinants of disease occurrence in dog populations at human-wildlife interfaces are unknown, and the assessment of risk factors and epidemiological parameters related to viral pathogen prevalence has rarely been performed in South American dog populations, except for some studies from Chile [9, 25]. These aspects have importance for disease prevention or control management because of the increasing presence of free-roaming dogs inside protected areas, enhanced dog-wildlife interaction, and the likelihood of context-dependent impact of disease circulation in different scenarios [1, 3].

Therefore, the aims of our study are to detect the presence, prevalence and risk factors associated with the exposure to viral agents relevant to carnivore conservation in populations of domestic dogs living in rural landscapes around remnants of the Atlantic Forest. This endangered biome is located in the most populous region of Brazil. Many endangered species, including carnivore mammals, depend on this highly fragmented ecosystem and occupy small areas surrounded by rural landscapes. Here, disease exposure in domestic dogs must be associated with poor management, free-roaming behaviour and other epidemiologically relevant features. A list of individual and environmental factors associated with previous exposure to viral pathogens is presented, highlighting the need for management actions for the improvement of health of dog populations and the urgent prevention of disease-induced mortality of already threatened Atlantic Forest’s wild carnivores.

## Methods

### Ethics and consents

Sampling was performed under permission from the household head or other responsible person. Required licenses were obtained from the State Forest Institute – IEF (UC: 080/10, 081/10 and 082/10). The study was approved by the Ethics Commission on the Use of Animals of the Pontiphical Catholic University of Minas Gerais (CEUA, PUC Minas 037/2010). Regarding the collection of data from humans and households, our project was examined by the Ethics Research Committee (Comitê de Ética em Pesquisa) of the Pontiphical Catholic University of Minas Gerais (PUC-Minas). A term about the confidential character of the records was read in every household. Animal manipulation procedures adhered to the guidelines from the COBEA (Brazilian College of Animal Experimentation) and the Animal Ethics Committee of FIOCRUZ (Oswaldo Cruz Institute Foundation).

### Study sites

**Fig. 1:**
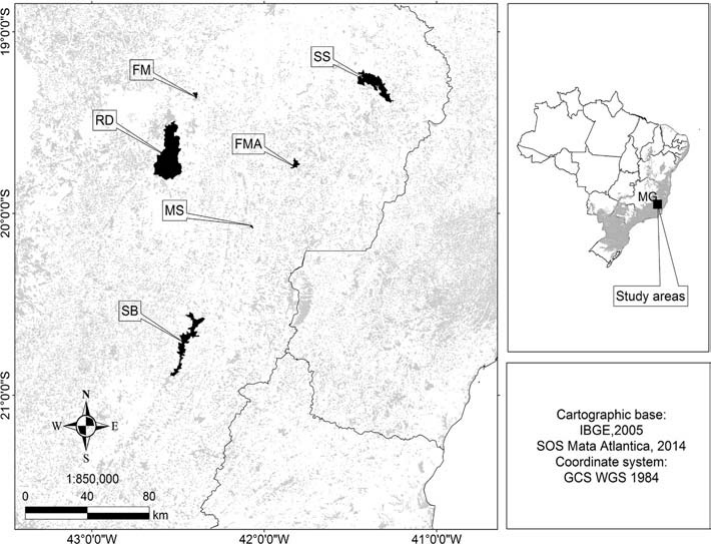
Location of the study areas. SB: Serra do Brigadeiro State Park; SS: Sete Salões State Park; RD: Rio Doce State Park; FM: Fazenda Macedônia Private Reserve; FMA: Feliciano Miguel Abdala Private Reserve; MS: Mata do Sossego Private Reserve (from Massara *et al*. PloS One 2015, 10(11): e0141333)

We selected rural households located at less than two kilometres from borders of six protected areas in the remnant Atlantic Forest of the state of Minas Gerais, south-eastern Brazil. These areas were selected for the presence of a protected fragment surrounded by human-dominated agricultural matrices, and were divided for analyses into two size classes, comprising three state parks (henceforth referred as large areas): Serra do Brigadeiro (PESB, municipality of Araponga), Sete Salões (PESS, municipality of Santa Rita do Itueto), and Rio Doce (PERD, municipality of Dionísio), and three private reserves (henceforth referred as small areas): Fazenda Macedônia (RPPNFM, municipality of Ipaba), Feliciano Miguel Abdala (RPPNFMA, municipality of Caratinga), and Mata do Sossego (RPPNMS, municipality of Simonésia) (see Fig. 1 and Table 1). Several wild carnivore species were recorded in the areas, including wild canids such as the crab-eating fox (*Cerdocyon thous*) and the maned wolf (*Chrysocyon brachyurus*), felids such as the puma (*Puma concolor*) and small wild felids (*Leopardus* spp.), mustelids (*Eira barbara*, *Gallictis cuja*), and procyonids (*Nasua nasua*, *Procyon cancrivorous*). According to a concomitant camera-trap study, free-roaming domestic dogs, mostly those living in surrounding rural properties, are frequently visiting and actually occupying the interior of these areas [26]. They live as human-subsidized mixed-bred free-ranging dog (host) populations distributed in small groups across the households and rural properties.

**Table 1:** Human and dog demographic characteristics at rural settlements around six protected areas of the Atlantic Forest of Minas Gerais State, southeast Brazil

Area (size in ha)	Distance from city (km)	Houses	Humans	Dogs	Dog:human ratio	Dogs per household
RPPNFM (3,343)	0.3	25	89	98	1.101	3.920
PESB (15,015)	3.3	31	125	86	0.688	2.774
PESS (13,370)	4.7	25	82	53	0.646	2.120
RPPNFMA (1,312)	10.5	18	53	60	1.132	3.333
RPPNMS (392)	7.7	25	102	49	0.480	1.960
PERD (36,100)	11	20	87	34	0.390	1.700
Total	-	144	538	380	0.706	2.638

### Questionnaire survey and blood sampling

Visits to the households, owner interviews and dog sampling were performed between January 2011 and August 2012. Every dog present at the households was aimed for data collection. Overall 320 dogs older than two months were sampled in 144 rural households. After permission from the owners, blood was collected under physical restraint from the jugular vein and a complete clinical examination of the dogs was performed by a veterinarian. A standardized questionnaire survey was administered to each owner. Factors related to animal management and behaviour that might be directly or indirectly associated with the exposure to viral agents were recorded for each household: number of dogs, mobility of dogs, access of dogs to the forest and villages, observed interactions between dogs and wildlife, recent dog disease or death, previous anti-rabies and multiple vaccination, veterinary assistance and the number of people. Individual and clinical features of dogs (sex, age, breed, sterilization, body condition, clinical alterations), which may influence behavioural patterns and pathogen exposure, were also obtained. Data were recorded in individual dog and per household files. Age of dogs was estimated through owner’s information matched with dental development observation. Body condition of dogs was scored from 0 (extreme emaciation) to 5 (extreme obesity). Refusals to the survey happened in six households, when the responsible person was absent.

### Serological testing

Blood samples were allowed to clot for at least two hours at room temperature. Serum was extracted after centrifugation in the field, and stored at −20 °C until sent to the laboratory for antibody detection and titration through duplicated serological testing for canine parvovirus (CPV, hemagglutination inhibition, 1:20 dilution as cut-off point), canine distemper virus (CDV, serum neutralization, 1:8 dilution as cut-off point), canine coronavirus (CCV, serum neutralization, 1:2 dilution as cut-off point), and canine adenovirus type-2 (CAV, serum neutralization, 1:16 dilution as cut-off point). Cut-off points were set according to previous literature, and aimed to maximize the sensitivity of the tests [27–30]. Prevalence is referred henceforth as the proportion of animals with detectable antibodies for each pathogen and considered as an indicator of previous pathogen exposure in dogs. Titres are expressed here as the inverse of the highest positive dilution. Higher antibody titres may reflect more recent infections, larger antigenic burdens (i.e. exposure to higher viral loads) or continued exposition, but also stronger individual immune responses to exposure, which are also dependent on many factors including nutrition, stress and genetics.

### Statistical analysis

Prevalence proportion ratios between grouped small versus large areas were compared through Yates-corrected chi square tests for each pathogen detected. Animals previously multiple-agent vaccination (*n =* 19) were excluded of the analysis to assess only natural viral circulation. Sex, breed, sterilization, mobility of dogs, access of dogs to the forest and villages, observed interactions between dogs and wildlife, recent dog disease or death, veterinary assistance and area size class (small and large areas) were used as binary or dummy variables. The continuous variables were age, body condition, number of dogs, and the number of people (which may act as fomites, for instance, for CDV and CPV [31, 32]) per household. Thus, fifteen variables were initially screened through univariate logistic regression tests with exposure status for each pathogen detected as the response variables.

All variables with a univariable test value of *P <* 0.3 were considered for subsequent inclusion in multivariable generalized linear mixed models (GLMM) with the exposure status for each pathogen as the response variables [33, 34]. Models were built with a manual backwards stepwise approach. Variables with lower P values were retained in the models until their exclusion resulted in a significant difference between subsequent models (*P <* 0.05). To select the best models we used the Akaike Information Criterion (AIC) values, and chose as candidate models those with ΔAIC < 2 in relation to the model with lowest AIC. We accounted for site/area variation in the data by including area as a random factor in all models. Chi-square tests were performed with the software Bioestat 5.0, while logistic regressions and GLMM’s were performed with the software R. We used the Strengthening the Reporting of Observational Studies in Epidemiology (STROBE) statement [35] as a guideline to report our data.

## Results

### Dog population and management traits

Relevant characteristics of sites, dog and human populations and the number of households sampled in each study area are described in Table 1. Males comprised 63.5 % (209/320) of dogs, thus sex ratio is male biased (1.88 males for each female). Only 21 dogs (6.5 %) are sterilized. Mixed bred dogs comprise 79.3 % (254/320) of the samples. Most dogs (78.4 %) are adult, with mean age of 3.3 years (39 months; range 3–216; mean 39.9 ± 36.05). Body condition scores are low in general (range 0.5–4, mean 2.1 ± 0.57).

Most dogs are allowed to roam freely, and only 10 % (33/320) live in restricted spaces as fenced or leashed dogs. Most dogs are reported to access near forests (249/320 or 77.8 %), and 30 % (96/320) have access to villages or small urban centres. Dog mortality or clinical disease in previous two years is reported by owners of 43 % and 31 % of dogs, respectively, but only four dogs show clinical symptoms compatible with viral disease (diarrhoea and ocular secretion) at the time of collection. Anti-rabies vaccination was performed in 85 % (261/320) of dogs, but multiple-disease vaccines (protective for the pathogens studied here) were applied in only 6 % (19/320) of dogs. Most dogs (53 %; 170/320) sampled are reported as having interacted with some wildlife species. Only 25 dogs (8 %) receive veterinary assistance throughout their lives. Most owners (63 %) feed their dogs with human leftovers, which were mostly protein-poor mixtures. Commercial dog food is provided in 39 %, and milk alone in 6 % of houses. In some households, combinations of commercial dog food plus milk (3.4 %) or leftovers (14 %) are used to feed dogs. Other items reported include milk whey and minced corn.

### Prevalence and titre frequency profile

Prevalence per study area is summarized in Table 2. Almost 85 % (320 of 380) of resident dogs were sampled. Antibodies against CPV, CDV and CAV were detected with a prevalence of 97 %, 15 %, and 27.8 %, respectively. Antibodies against CCV were not detected in our samples. The three former agents were detected in all six areas, except for CDV antibodies that are absent in dogs from RPPNFMA. According to chi square test results, CPV is more prevalent in small areas (173/174, 99 %) than in larger areas (138/146, 94 %) (*p =* 0.004). Accordingly, more CDV positive dogs are present in small than in large areas (33/174 or 19 %, and 15/146 or 10 %, respectively; *p =* 0.015). CAV prevalence do not differ between large and smaller areas (46/174 or 26 %, and 43/146 or 29 %, respectively; *p =* 0.27).

**Table 2:** Prevalence (in bold) for canine parvovirus (CPV), canine distemper virus (CDV), and canine adenovirus (CAV) in dogs sampled in the rural zone surrounding Atlantic Forest fragments in Brazil

Area	Dogs	Sampled	% sampled	CPV +	%	CDV +	%	CAV+	%
RPPNFM	98	84	85.7	83	**98.8**	25	**29.7**	28	**33.3**
PESB	86	67	77.9	65	**97.0**	11	**16.4**	15	**22.4**
PESS	53	47	88.6	47	**100**	2	**4.2**	9	**19.1**
RPPNFMA	60	49	81.6	49	**100**	0	**0**	11	**22.4**
RPPNMS	49	41	83.6	41	**100**	8	**19.5**	7	**17.1**
PERD	34	32	94.1	26	**81.2**	2	**6.2**	19	**59.3**
Total	380	320	84.2	311	**97.2**	48	**15**	89	**27.8**

**Fig. 2:**
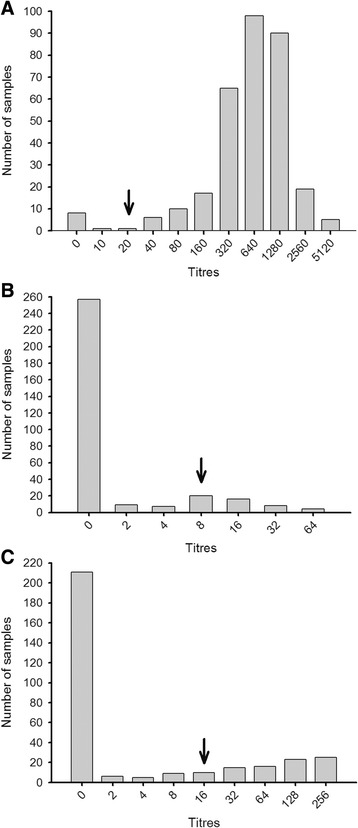
Titre frequency distributions for CPV (**a**), CDV (**b**) and CAV (**c**) in domestic dogs living around protected areas of the Atlantic Forest in Minas Gerais, Brazil (2011 to 2012). Cut-off points are indicated by arrows

Titre frequency distributions for the pathogens detected are depicted in Fig. 2. Most samples have high antibody titres for CPV (>160) and for CAV (>64), but for CDV most positive samples have relatively lower titres.

### Risk factor analysis

Associated variables with significance values below the fixed threshold (*P <* 0.3) in the univariate screening tests are listed in Table 3. Regarding multivariable analyses, the best CAV model included sex, age of dogs, number of people, interaction with wildlife, and access to villages as significant associations. However, sex, age, number of people and access to villages were present in all candidate models. CDV best model included age of dogs, body score, access to forest and number of people per household. Age and number of people were retained in all candidate models. For CPV, four variables were retained in the best model as significantly associated with exposure: dog mobility, recent dog disease, veterinary assistance, and area size class. Mobility and veterinary assistance figured in all candidate models. Results are summarized in Table 4.

**Table 3:** Univariate regression analysis results for variables associated with canine adenovirus (CAV), canine distemper virus (CDV) and canine parvovirus (CPV) status in unvaccinated dogs sampled in the rural zone surrounding Atlantic Forest fragments in Brazil

Pathogen/variables	Odds ratio	95 % CI	P value
CAV			
Sex (female)	1.617	0.927–2.90	0.09
Age	1.014	1.007–1.021	6.48x10^−5^
Body score	1.599	1.013–2.554	0.045
Number of people	1.13	0.95–1.359	0.16
Access to villages	3.806	2.221–6.575	1.3x10^−6^
Recent dog mortality	1.457	0.868–2.445	0.153
Interaction with wildlife	0.619	0.367–1.036	0.069
CDV			
Sex (female)	0.676	0.355–1.304	0.235
Age	1.009	1–1.016	0.024
Body score	1.756	1.001–3.11	0.05
Number of people	1.395	1.121–1.746	0.003
Number of dogs	1.120	0.996–1.252	0.048
Access to forest	3.190	1.225–10.927	0.033
Recent dog disease	1.501	0.763–2.883	0.22
Area size class	0.515	0.255–0.997	0.05
CPV			
Mobility	18.266	3.786–98.234	0.0002
Access to forest	5.092	1.09–26.42	0.036
Recent dog mortality	4.562	0.766–86.747	0.16
Recent dog disease	0.165	0.023–0.783	0.033
Interaction with wildlife	2.75	0.582–19.415	0.231
Veterinary assistance	0.076	0.015–0.41	0.001
Area size class	0.12	0.006–0.765	0.05

Only variables with significance below the threshold (*P <* 0.3) are shown

**Table 4:** Generalized linear mixed modelling of factors associated with canine adenovirus (CAV), canine distemper virus (CDV) and canine parvovirus (CPV) exposure status of unvaccinated dogs sampled in the rural zone surrounding Atlantic Forest fragments, Brazil

Model	Variables	AIC	Δ AIC
*CAV*	Null	349.00	
Model 6	Age + city	315.73	3.71
Model 5	Age + people + city	314.46	2.44
**Model 4**	**Sex + age + people + city**	**312.78**	**0.76**
**Model 3** ^a^	**Sex + age + people + city + fauna**	**312.02**	**0**
**Model 2**	**Sex + age + people + city + death + fauna**	**312.23**	**0.21**
**Model 1**	**Sex + age + score + people + city + death + fauna**	**312.98**	**0.96**
*CDV*	Null	241.00	
**Model 7**	**Age + people**	**232.13**	**1.10**
**Model 6**	**Age + people + forest**	**231.29**	**0.26**
**Model 5** ^a^	**Age + score + people + forest**	**231.03**	**0**
**Model 4**	**Sex + age + score + people + forest**	**231.92**	**0.89**
Model 3	Sex + age + score + people + forest + size	233.69	2.66
Model 2	Sex + age + score + people + forest + sick + size	235.23	4.2
Model 1	Sex + age + score + people + dogs + forest + sick + size	237.23	6.2
*CPV*	Null	65.70	
**Model 6**	**Mob + vet**	**56.15**	**1.34**
**Model 5**	**Mob + sick + vet**	**54.82**	**0.2**
**Model 4** ^a^	**Mob + sick + vet + size**	**54.80**	**0**
Model 3	Mob + forest + sick + vet + size	56.53	2.27
Model 2	Mob + forest + death + sick + vet + size	58.36	3.56
Model 1	Mob + forest + death + sick + fauna + vet + size	60.32	5.51

Models with the lowest AIC values were considered as best fit (^a^) and models with Δ AIC < 2 were considered as candidate models (in bold)

Score = body score, people = number of people, city = access to villages, death = recent dog mortality, fauna = interaction with wildlife, dogs = number of dogs, forest = access to forest, sick = recent dog disease, size = area size class, mob = mobility, vet = veterinary assistance

## Discussion

Frequent contact with domestic dogs increases the exposure and disease risk for wild carnivores [8, 36]. Therefore, local pet management practices allowing dog’s predominantly free-roaming habits, poor veterinary assistance, along with recent dog death and disease reports and the low multiple vaccination coverage detected *per se* place the wild carnivores at the study sites in a potentially dangerous scenario of disease spill over (or spillback) from dogs.

### Exposure patterns and antibody titre profile

Exposure to most pathogens tested is widespread throughout the study sites, and prevalence is widespread and moderate to high, particularly for CPV. It must be noted that CDV and CPV have higher fatality rates than do CAV and CCV milder infections [31, 32, 37]. Fatality rate may impact prevalence by removing exposed individuals from the population and this can result in the low prevalence observed here, for instance, for CDV. Also, prevalence of CDV might be low in these areas because a ‘wave of infection’ could have passed through the area in the past, and recent circulation of this virus may not have occurred regularly [9].

Regarding antibody titre frequency and duration of immunity, mostly high levels of antibodies against CPV and CAV were found. The duration of antibodies to the viral agents studied here is longer than two years [38], and such titres may indicate that the exposure to these agents is mostly recent. However, as said before, it is not possible to accurately determine the timing of exposure from antibody titres, and our cross-sectional serological approach does not permit deeper inferences on temporal-spatial dynamics of exposure neither detects pathogen introduction or seroconversion events.

### Prevalence comparisons between small and large protected areas

Proportionally more CDV and CPV-exposed dogs were found in small areas. Perhaps the reduced perimeter of these areas allows less space between properties and households, and ensures higher host densities, contact rates and exposure (including environmental) to these agents. Therefore, small areas should be prioritized in health improvement efforts for rural dogs aiming also to prevent wildlife disease-associated mortality in the Atlantic Forest, which is currently mostly composed by relatively small remnants [39].

### Risk factor modelling

Of fifteen initially assessed variables, thirteen entered multivariate tests, and eleven remained as significant associations with exposure for at least one pathogen in the best models: sex, age, body score, mobility, veterinary assistance, number of people per household, interaction with wildlife, recent disease in dogs, area size class and access to villages and forests. However, one must account for associated bias due to issues with missing controls in an observational prevalence study when interpreting data for risk factor detection, and there might be biologically relevant putative causalities out of the full set of factors presented. Moreover, the statistical associations found do not necessarily imply direct causation.

### Canine parvovirus

High levels of circulation indicated by the high prevalence of exposure indicate that CPV is perhaps one of the most dangerous agents in this scenario. It is one of the most commonly reported canine viral agents worldwide [3, 31], including in South American wild canids (e.g.) [16, 18, 19]. High prevalence was concomitantly observed in wild and domestic canids from two protected areas [18, 19] in the same state of the present study, although in the Cerrado Biome. CPV infection has proven capable to cause serious population impacts (mostly through pup mortality) in wild carnivores, for instance, grey wolves (*Canis lupus*) [6]. However, our cross-sectional approach does not distinguish epidemic from endemic states. For instance, a CDV epidemic that might blow through these mostly susceptible dog populations might also cause high dog mortality and interspecific transmission that can be dangerous for wild carnivores as well.

The modelling shows that unassisted dogs that roam freely have more chances of exposure for CPV. This is explained by higher contact rates with diseased animals and enhanced environmental exposure due to the great environmental resistance of this virus [31], therefore dog restriction must be enforced around protected areas. Dogs that never received veterinary care were also more prone to exposure, and this emphasizes the role of veterinarians in health promotion and maintenance at human/wildlife interfaces. Other factors appearing in candidate models (recent dog disease and area size class) indicate that CPV may be causing significant morbidity in the study sites, and that small reserves require priority for the control of the disease.

### Canine distemper

Canine distemper is a systemic highly fatal disease, representing a major conservation concern around the globe [3, 32]. Evidence of infection in dogs is widespread in and around South American protected areas [18, 20, 22, 23]. Antibodies against distemper were already found in Brazilian wild felids and canids (e.g.)[17, 19], and there are reports of distemper-induced mortality in two Brazilian fox species, the crab-eating fox *C. thous* and the hoary fox *Lycalopex vetulus* [14, 15]. In Chile, domestic dogs have proven blamed for the transmission of CDV to wild canids [9]. However, CDV transmission may be, in some cases, predominantly maintained by wild reservoirs [4, 32, 36].

In our rural settings, age, body score, number of people in the household and access to forests were mostly associated with CDV prevalence. However, only age and the number of people appeared in all candidate models. Older animals might have had more chances of exposure events throughout their lives. However, since CDV is a highly lethal disease [32], the low prevalence and the age effect observed suggest that CDV seropositive animals may be survivors of a past wave of infection in some of the study areas. The number of people cohabiting households was positively associated with CDV seropositivity. This raises the possibility of fomite transmission, mentioned in the literature as a mode of spread of this highly infectious virus which is mainly transmitted by contact or aerosols [32]. Male dogs may be at higher risk of exposure, probably because of their more aggressive and roaming behaviours [40]. Higher body scores may also favour dog free-roaming behaviours and ensuring both exposure and antibody response to the virus, which, according to the presence of the access to forests among risk factors, might be occurring when dogs walk inside forested areas. As said before, CDV can be maintained in wild reservoir species alone [4, 36].

### Canine adenovirus

Mostly unstudied, CAV is also capable to cause damage to wildlife populations [3], although its impact is still unknown. This directly transmitted virus may cause severe respiratory disease being of concern for domestic dog health, and evidence of exposure was found in many wild species [37] including in South American wild carnivores and sympatric dogs from Bolivia [16, 23] and Brazil [18, 19].

For CAV, female sex, age, number of people and access to villages entered all selected models. Females are more exposed to CAV, and this is probably related to behavioural differences, as females tend to display less roaming and aggressive behaviours [40], and thus, spend relatively more time around their homes in rural and urban settings, leading to host aggregation and enhancement of the density-dependent transmission of CAV. The explanation for increasing prevalence with age is that older dogs probably had moved more through time and faced more opportunities for contact and exposure to CAV, and this is expected since older animals tend to have more opportunities of infection when disease-induced mortality is low or infections are often fatal [40]. The association with the number of people in the households reinforces the questioning about the fomite spreading of canine viruses in rural environments. However, this is not mentioned as a CAV transmission mode [37]. The access to villages was a strongly associated factor, appearing in all candidate models. Urban settings hold higher-density dog populations [1], what enhances the maintenance and transmission of the density-dependent CAV [37]. Therefore, such freedom of rural dog movement must be prevented in order to reduce exposure and infection by CAV in near urban areas and subsequent spillover to wild species. Recently, data from the same state showed that free-roaming behaviours (e.g. hunting) are among risk factors for canine neosporosis [11]. Additionally, recent mortality in dogs may be associated with CAV infection in the study sites. Dogs with higher body scores may have survived past infection waves and sustained the mostly low antibody levels found. Interactions with wildlife may also be increasing the risk for CAV. However we cannot infer whether the main reservoirs are domestic or wild animals. Nevertheless, the free-ranging behaviour of dogs must be inhibited through fence or leash restraining in order to reduce contact rates and opportunities for general pathogen exposure and transmission in rural/wild interface areas.

### Preventive aspects

Although we did not test the samples against rabies, our survey revealed that despite the apparently good previous vaccination coverage (more than 80 %), several owners reported the total absence or the periodic lack of visits of health agencies promoting vaccination against rabies in their households in some years. Canine-mediated rabies is a multi-species highly fatal disease, representing a major problem for carnivore conservation particularly in Africa [3]. In Brazil there are domestic animal rabies control programmes through vaccination since the 1980’s, and reports of wildlife mortality have been attributed to the disease. Additionally, serological evidence of exposure was already found in Brazilian carnivore species [41]. Therefore, more attention should be given to rabies in wildlife-domestic animal interfaces, and the vaccination programme should be reinforced so as to continuously warrant good coverage in these areas.

In our scenario, dogs with a history of vaccination against agents other than rabies, which are not cost-free, may be an indicator of increased owner care. Better care for the dog would result in better physical and immunologic condition, and better supportive care and veterinary care in times of illness. Those healthier animals will be more likely to survive an illness, and a higher survival rate would lead to a greater proportion of seropositive dogs. Unfortunately, the mostly unvaccinated dogs in these populations may be less likely to receive a high level of owner care. Lack of owner care may result in lower probability of supportive or veterinary care when ill, and an overall decrease in animal health. Poor health and lack of veterinary care would result in a higher death rate in the result of an illness. A higher death rate would remove exposed animals from the population. Therefore, unvaccinated dogs may have less supportive care from owners, which would lead to higher death rates in the face of illness, which would lead to artificial decline in the measured population proportion of seropositives, as may be the case for CDV in this study. However, our data set does not permit such distinction, and we acknowledge the uncertainty about these relationships.

Commercial multiple vaccines against CDV, CPV, CAV, CCV and other canine pathogens are available in the region. However, owner unawareness added to the relatively prohibitive costs of vaccines, and the fact that such intervention may seem directed solely to protect the health of dogs make multiple-agent vaccination of dogs a lesser priority for the mostly low-income rural families, as shown by the low percentage of vaccinated dogs in this study. We are unaware of the use of other possible cross-reactive vaccines, such as those using adenovirus as a vector, in the area. This means that multiple vaccination have to be reinforced, even though with a more flexible and viable interval in these areas [38], if the aim is to induce protective herd immunity against other dangerous pathogens in dogs from wildlife-rich areas. Vaccination schemes for dogs around protected areas, directing to protect wild carnivore and human welfare has proven successful [42], notably with concomitant low coverage vaccination of wildlife species [5]. In our case, the dog population living in proximity of protected area borders should be targeted in comprehensive multiple-agent vaccination schemes. Vaccination of dogs can improve the collective immune status or herd immunity necessary to avoid or decrease the transmission of some agents. However, as pointed out by some authors, vaccination of naturally exposed populations may not be of great value for highly prevalent pathogens [10], and this may be the case for CPV in our study area. Nonetheless, the transmission of lower local prevalence pathogens such as CDV and CAV may be successfully limited by dog vaccination in this case. Decreasing dog numbers is also necessary to keep the population below transmission thresholds of most directly transmitted canine pathogens, and this can be achieved through sterilization and education campaigns. According to our results, prohibiting dogs from moving across the interface and spreading or acquiring pathogens is essential for disease prevention, and responsible ownership reinforcement alongside with legal penalties for irresponsible owners is highly recommended. Health monitoring should, afterwards, be continuously performed in both domestic and wildlife species, to assess the efficacy of the proposed measures.

## Conclusions

This study represents the first attempt to detect pathogens of concern for carnivore conservation in dogs living in rural settlements around Atlantic forest fragments, and to reveal associated factors that can be managed to improve domestic dog’s health and consequently protect wild carnivores from disease-induced population declines in these areas, even though local wildlife health status is unknown. Fortunately in this case, interventions should be directed to the human component of the system. The management involving human behaviour related to domestic animal management is more easily tractable [43] and might decrease successfully dog-to-dog and dog-to-wildlife disease transmission without relevant side effects. Some of the risk factors shown are linked to poverty, which in turn is associated with poor domestic dog health [44]. Therefore, programs involving the improvement of life quality for local human populations may warrant better domestic animal care and health. It also meets conservation goals to reduce unwanted dog-wildlife interactions such as predation, competition and harassment [1, 2]. Restriction of dog space and movements, control of the reservoir population through sterilization, and proper vaccination programmes are among required measures for the purpose. Finally, the study have generated some interesting hypothesis that can be further tested, and provided a confirmatory set of information that enhances the understanding of natural viral exposure patterns in rural (and mostly free-roaming) dog populations and of processes linked to disease transmission at human-domestic animal-wildlife interfaces.

## Abbreviations

CAV: canine Adenovirus
CCV: canine Coronavirus
CDV: canine Distemper Virus
CPV: canine Parvovirus
PERD: Rio Doce State Park
PESB: Serra do Brigadeiro State Park
PESS: Sete Salões State Park
RPPNFM: Fazenda Macedônia Private Reserve
RPPNFMA: Feliciano Miguel Abdala Private Reserve
RPPNMS: Mata do Sossego Private Reserve
STROBE: strengthening the reporting of observational studies in epidemiology
